# Facilitating permeability of landscapes impacted by roads for protected amphibians: patterns of movement for the great crested newt

**DOI:** 10.7717/peerj.2922

**Published:** 2017-02-28

**Authors:** Cátia Matos, Silviu Petrovan, Alastair I. Ward, Philip Wheeler

**Affiliations:** 1 School of Environmental Sciences, University of Hull, Hull, North Yorkshire, United Kingdom; 2 Centre for Environmental and Marine Sciences (CEMS), University of Hull, Scarborough, North Yorkshire, United Kingdom; 3 Froglife, Peterborough, United Kingdom; 4 National Wildlife Management Centre, Animal and Plant Health Agency, York, North Yorkshire, United Kingdom; 5 School of Environment, Earth and Ecosystem Sciences, Open University, Milton Keynes, United Kingdom

**Keywords:** Connectivity, Dispersal, Great crested newt, Migration, Smooth newt, Underpass, Wildlife crossing, Road ecology

## Abstract

Amphibian populations are highly vulnerable to road mortality and habitat fragmentation caused by road networks. Wildlife road tunnels are considered the most promising road mitigation measure for amphibians yet generally remain inadequately monitored, resulting in mixed success rates in the short-term and uncertain conservation benefits in the long-term. We monitored a complex multi-tunnel and fence system over five years and investigated the impact of the scheme on movement patterns of two newt species, including the largest known UK population of the great crested newt (*Triturus cristatus*)*,* a European Protected Species. We used a stage descriptive approach based on capture positions to quantify newt movement patterns. Newt species successfully used the mitigation but the system constituted a bottleneck to movements from the fences to the tunnels. Crossing rates varied widely among years and were skewed towards autumn dispersal rather than spring breeding migration. There was a substantial negative bias against adult male great crested newts using the system. This study indicates that road tunnels could partially mitigate wider connectivity loss and fragmentation at the landscape scale for newt species. However, the observed bottleneck effects and seasonal bias could have population-level effects which must be better understood, especially for small populations, so that improvements can be made. Current requirements for monitoring mitigation schemes post-implementation are probably too short to assess their effectiveness in maintaining connectivity and to adequately understand their population-level impacts.

## Introduction

Worldwide, road networks represent a major threat to amphibian population viability. Roads restrict and interrupt amphibian movements and cause high mortality through road kills during seasonal migration and dispersal (Fahrig et al., 1994; Hels & Buchwald, 2001; Glista, DeVault & DeWoody, 2007; Matos, Sillero & Argaña, 2012; Petrovan & Schmidt, 2016).

Road crossing structures for amphibians, typically small diameter tunnels or underpasses and associated fence systems, have been implemented for over 30 years along spring migration routes in Europe and are currently considered the most promising road mitigation solution for amphibians (Brehm, 1989; Iuell et al., 2003; Lesbarrères & Fahrig, 2012). However, while some studies report reductions in road mortality rates, at least in the short term, few have performed a detailed comparative analysis of tunnel- and fence-use by amphibians (Jochimsen et al., 2004; Pagnucco, Paszkowski & Scrimgeour, 2012) and virtually none for newts (Schmidt & Zumbach, 2008; Beebee, 2013).

Successful and robust mitigation is especially relevant for declining or threatened species where road networks could severely impact on the population connectivity and ultimately long term population survival. The great crested newt (*Triturus cristatus*) is a European Protected Species which has declined substantially over recent decades, largely due to habitat loss and habitat degradation (Langton, Beckett & Foster, 2001; Jehle, Thiesmeier & Foster, 2011). However, the species remains relatively widespread in the UK, including in semi-urban environments, and is therefore regularly the subject of road mitigation schemes in an attempt to maintain habitat and population connectivity between the two sides of the road. Such schemes can involve populations of hundreds or even thousands of individuals, carrying substantial financial costs and cause significant delays for infrastructure projects. Evaluation of mitigation success is therefore required to ensure that only sound practices are employed (Ward, Dendy & Cowan, 2015).

Road connectivity schemes typically adopt linkage strategies which target species dispersal as the main process determining landscape-scale connectivity (Baguette & Dyck, 2007; Baguette et al., 2013). For pond-breeding amphibians, such as *T. cristatus*, metapopulation dynamics are highly dependent on connectivity and consequently dispersal as determining fundamental processes for long term population viability (Halley, Oldham & Arntzen, 1996; Semlitsch, 2008; Griffiths, Sewell & McCrea, 2010). In addition, barriers to movement may limit individuals’ ability to secure specific habitat requirements at different stages of maturity (Sinsch, 1990). Adult movements (migration) between aquatic and terrestrial habitats are defined as short-term migration movements because of their duration and distance (Pittman, Osbourn & Semlitsch, 2014). Long-term, wide-ranging movement (dispersal) is primarily performed by juveniles, which move significantly more among sub-populations and through landscapes than adults (Rothermel, 2004). Therefore, temporal and spatial variation in amphibian movements should be incorporated into assessments of the effectiveness of road crossing structures and mitigation schemes (Clevenger & Waltho, 2005). Equally, most published road mitigation studies have only presented use-frequency over short time periods (1–2 years), and lacked comparisons regarding seasons and trends over several years (Jackson & Tyning, 1989; Allaback & Laabs, 2003; Pagnucco et al., 2011).

We performed a 5-year monitoring study aiming to assess potential functional connectivity of a road mitigation scheme for *T. cristatus* and other amphibian species in the UK. We investigated whether newts successfully crossed the road using the mitigation scheme and if crossing rates differed between species, sexes and age classes. We hypothesised that use of the mitigation scheme by newts would be greatest during seasonal peaks of activity (autumn and spring), independently of age, and would increase over time as the vegetation around the tunnels became better established. Finally, we investigated if newt movement was facilitated by the tunnels between the two parts of the population separated by the road. The main objectives were to: (1) characterise different types of newt movement for age and sex class in relation to the mitigation system, (2) assess annual, seasonal and spatial differences in movement patterns and (3) evaluate if movement through the tunnels was maintained over time by determining which variables explain seasonal and directional movement variance among years of monitoring.

Ultimately, our goal was to understand how the mitigation scheme supported the movements and connectivity of the newt population and therefore draw conclusions on its effectiveness for the maintenance of the wider population in the long term. Given that no published data exist on road mitigation systems for *T. cristatus* this study could inform other current and future mitigation schemes for this protected species and newt species in general.

## Material and Methods

### Study area

**Figure 1:**
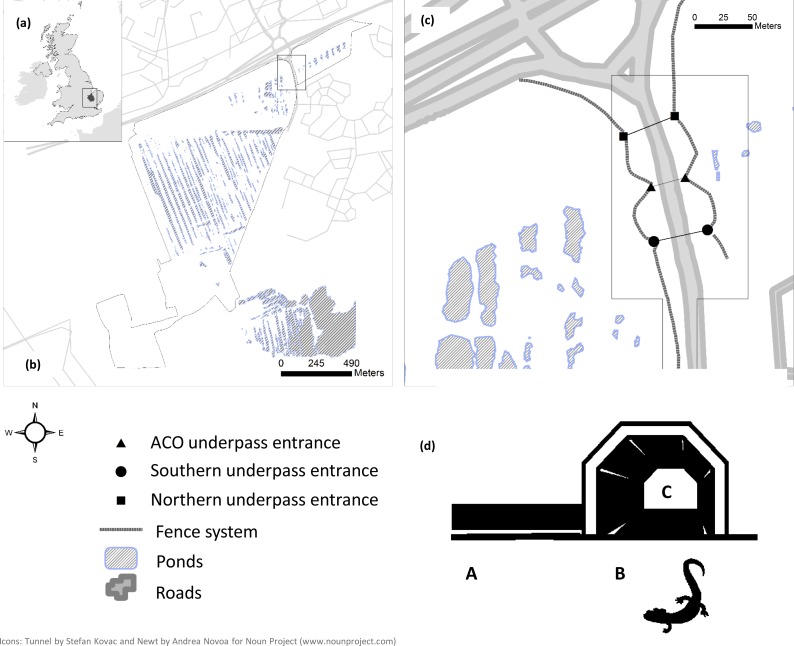
(a) Location of the study area in Peterborough, UK (b) west (large) and east (small) sections of Hampton Nature Reserve (SSSI) (c) monitored sections of the mitigation system (in bold) (d) For each capture point a code was defined: movements along the fence system (A); movements in tunnel/attempted crossing (B); movement in tunnel/successful crossing (C).

The study was conducted in Orton Pit/Hampton Nature Reserve (52°32′24N, 0°16′53W), a designated Special Site of Scientific Interest, Special Area of Conservation and Natura 2000 site, located south of Peterborough, Cambridgeshire (UK) (Fig. 1A). This 145 ha reserve mainly comprises a section of former industrial brick clay extraction site but also includes woodland and patches of scrub. The main site is characterised by a complex of over 340 ponds, ranging from 15–50 years old. Between 1990 and 2000 a large-scale habitat restoration took place including pond modification and fish eradications. Concomitantly, an extensive amphibian translocation programme took place with 54,000 adult amphibians and 66,000 juveniles moved to the reserve from the nearby brickpit area. Of these, 24,000 were adult great crested newts and 9,000 were adult smooth newts (*Lissotriton vulgaris*) with the rest represented by common toads (*Bufo bufo*) and common frogs (*Rana temporaria*) (HCI, 2000). Following translocation, concrete ‘newt barriers’ were installed on sections of the reserve along the border of the new development land and associated road. The site is currently home to potentially the largest single population of great crested newts in the UK and possibly Europe, estimated at around 30,000 individuals, as well as a very large population of smooth newts, but common frogs and common toads have become exceedingly rare (Froglife, 2012a; Froglife, 2012b).

The northern reserve area is divided by a 10–12 m wide, high-traffic-volume (1,000–10,000 vehicles/day) road constructed in 2006 which serves the new housing development (Fig. 1B). Construction of this road involved the reprofiling of some large ponds and filling in of others. Consequently, a comparison of pre-road amphibian movement rates across the pre-road and post-road was not possible because of changes in the landscape and the lack of specific monitoring data before the road was built. The road mitigation system was composed of: one polymer concrete ‘amphibian tunnel’, with open slots at the top, manufactured by ACO Germany (0.5 m diameter, 30 m long), two large ARCO concrete and metal sheet underpasses (5.5 m wide × 2 m high, 40 m long) spaced 100 m apart, and two heavy duty plastic fence systems (Herpetosure UK, 200–300 m in length), placed 10–50 m away from the road, angled to guide amphibians towards the tunnels. In an effort to minimise human disturbance the entrances of the large tunnels were protected with a locked bar fence and gate system. The three underpasses (northern, central or ACO, and southern tunnel) connect the two main sections (west and east sides of the road) in the north of the reserve (Fig. 1C).

### Field methods and amphibian movement patterns

Surveys were conducted between April 2007–October 2012 with no data collected in 2009. Monitoring averaged 7 months per year and 8–12 days per month, starting in spring (typically March) and ending in autumn (mid-late October), with no monitoring during winter due to amphibian inactivity. Tunnel usage was monitored using pitfall traps at both entrances of the north and south tunnels. Tunnel pitfalls, extended across the tunnel width, had double (inner and outer) 25 cm deep concrete and metal sheet trenches, each with an inverted top edge. This allowed the recording of complete and attempted crossings in these two tunnels: newts trapped in the inner trench travelled through the tunnel (completed), those in the outer trench just entered the tunnel (attempted). In addition, bucket pitfall traps and a temporary plastic fence were used for monitoring the central ACO tunnel. Inner/outer trenches were opened for the same number of times on each side, rotating every 4 days. The total number of trapping days varied in the first two years as the methodology was tested (Table 1). Trapping focused on spring and autumn, the main periods of amphibian movements, but included at least 4 days of trapping per month during the summer months. From 2008 until the end of the study the fence system was surveyed on trapping nights using night-time torch surveys. Additionally, in 2008 only, short drift fences and three 10-l plastic bucket pitfall traps were placed on each side of the road in front of the tunnel fences (east and west). Traps were checked daily, in early morning and again in the evening along with fence checks.

**Table 1:** Response and predictor variables used for the GLMM analysis.

Code	Variable description	Values (mean ± SE)
MI_*s*_	Movement index for season. Number of observations (amphibian captures), per capture point (A, B and C) per time period (number of night surveys) in each season (autumn, spring and summer). Continuous variable.	0.29 ± 0.06 (0–3.78)
MI_*d*_	Movement index for direction. Number of observations (amphibian captures), per capture point (A, B and C) per time period (number of night surveys) in each side of the road (East and West). Continuous variable.	0.12 ± 0.03 (0–1.72)
Age	Amphibian age classes. Factor.	Adults, Juveniles
Season	Monitoring seasons. Factor.	Autumn, spring and summer
Side	Side of road where amphibians were observed moving/captured. Factor	East, west
Type	Type of movement in the mitigation system. Capture points. Factor.	Moving along the fence (A), Attempt cross at tunnel entrance (B) and sucessful crossing in the tunnel (C).
Species	Newt species. Factor	*Triturus cristatus* (TC) and *Lissotriton vulgaris* (LV)
Year	Years of monitoring. Factor	2008, 2010, 2011, 2012

Amphibian species, number, sex and age class (adult or juvenile) were recorded together with the position in the mitigation system (tunnel/fence) and side of the road. No individual marking was performed and trapped amphibians were released in vegetation near the capture point. All trapping and handling was done under a Natural England licence (Ref: 04/01204/REM).

Although the tunnels were primarily put in place for the protected *T. cristatus*, which was also the main focus of the monitoring, *L. vulgaris* data were included at all stages during the analysis.

### Variables and data analysis

We coded newt movement on the basis of capture location within the mitigation scheme and their position in relation to the road (Fig. 1D). Captures along the fences were coded ‘A’, captures at tunnel entrances coded ‘B’, and captures of animals which had successfully crossed the road through tunnels coded ‘C’.

In 2007 the northern tunnel was mostly flooded and the additional fence system monitoring (to assess ‘A’ movements) was only started in 2008. Consequently, we only used data from 2008–2012 for this part of the analysis. Data were grouped into seasons: ‘spring’ (March, April, May); ‘summer’ (June, July, August); ‘autumn’ (September, October). Direction classes (‘east’ and ‘west’) describe (1) the position of capture with respect to the road such that animals captured at fences and tunnel entrances (A, B) and (2) for those that successfully crossed from east to west (C) would be classified as ‘West’ and similarly those that moved through tunnels from west to east were classified as ‘East’.

In order to standardise measures of movements among years, we developed an index (MI) that represents the amphibian use of the mitigation scheme at different observation points (A, B and C) and allows data to be compared separately for season and direction without bias due to differences in trapping effort each year:

\begin{eqnarray*}{\mathrm{MI}}_{ij}={n}_{i}/{\mathrm{CN}}_{j} \end{eqnarray*}

where *n*_*i*_ is the number of observations (amphibian captures separated by age class) of a species for each capture point *i* and CN_*j*_ is the number of capture nights per time period *j* (which varied for years (MI_*y*_), season (MI_*s*_) and direction (MI_*d*_)).

We estimated differences in captures between age (adults/juveniles), sex (male/female) classes and among capture points (A, B and C) using Pearson chi-square test (*χ*^2^). We examined the relative importance of age, season, side of capture, movement type and species for two movement patterns (seasonal and direction) along the years of monitoring (see Table 1 for details on variables). A generalized linear mixed-effect model (GLMM) with a zero-inflated Poisson (ZIP) error structure with log link was fitted for two response variables (MI_*s*_ and MI_*d*_), ZIP were used due to high presence of zeros in response variable distribution, this way potential overdispersion and bias is avoided in parameter estimation (Bolker et al., 2012). We separated the analysis into four models to clarify the role of each independent variable, considering years as a random effect.

Two null models (one for season and another for directionality) containing the most significant variables and intercept were included for comparison (age + season/side + type + species (1|year)). From here we tested three models with the most significant variables, with no test for interactions. We compared model parsimony using Akaike information criterion (AIC) to optimize goodness-of-fit but avoid overfitting of the candidate models (Burnham & Anderson, 2002). After selecting the most parsimonious model, we determined the significance of fixed factors by analysis of deviance (Burnham & Anderson, 2002).

All statistical procedures were carried out using R 3.2.2 (R Development Core Team, 2014). ZIGLMM models were fitted using glmmADMB package (Bolker et al., 2012).

## Results

A total of 831 amphibian captures were recorded over 353 trapping nights during the five years (Table 2). Fence and tunnel captures were highest in autumn (57.3%), spring (34.4%) and summer (8.3%). All four amphibian species found in the study area were recorded during surveys with the two anurans forming less than 1% of captures (*R. temporaria* 0.84%, *B. bufo* 0.12%). *T. cristatus* was the most frequently caught species (87.6% of captures) alongside *L. vulgaris* (11.4% of captures) (Table 2).

**Table 2:** Survey effort: number of survey days per year of monitoring and number and percentage of amphibian species recorded in the system per year.

Monitoring					Species				
Year	Months	Seasons	Days	Mean (days per month)	*N*	*T. cristatus*	*L. vulgaris*	*R. temporaria*	*B. bufo*
2007	5	2	48	9.6	10	6	3	0	1
2008	9	3	113	12.5	234	197	36	1	0
2010	8	3	64	8.0	248	209	36	3	0
2011	6	3	64	10.67	48	41	4	3	0
2012	8	3	64	8.00	291	275	16	0	0
**Total**	36	14	353	9.76	831	728	95	7	1
**%**					100	87.61	11.43	0.84	0.12

Age class was determined for 821 newt (98.8%) observations (Table S1). Adult *T. cristatus* represented 60.4% of the species captures versus 39.5% juveniles. By contrast, for *L. vulgaris* 69.9% of captures were juveniles and 30.1% adults. Sex was determined for almost all adult newts (i.e., 464 newts, Table S1) with *T. cristatus* adult females outnumbering adult males by over three to one (78.5% of captures).

There were higher numbers of detections along the fences than inside the tunnels (64.8% and 35.2% of captures, respectively) (Table S1). The short drift fences deployed in 2008 only captured 24 individuals (4.8% of the total individuals at the fence. The southern tunnel produced the highest number of newt captures (142 observations, 49%) followed by 125 in the northern tunnel and 23 in the central ACO tunnel (respectively with 43% and 8% of the records). More newts were captured on the main reserve side (‘West’, 60%) than on the east side of the road (40%).

**Figure 2:**
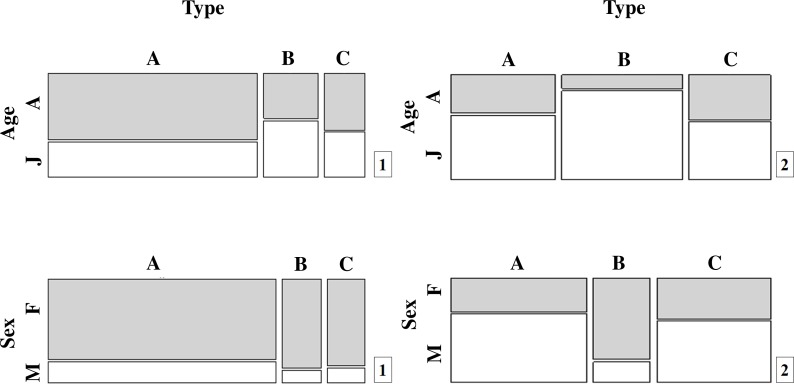
Population proportions for the different movement types (A, B and C) by age and sex for (1) *T. cristatus* and (2) *L. vulgaris*.

### Newt movement patterns in the road mitigation system

More *T. cristatus* and a higher proportion of adults were caught at the fence (A) than at tunnel entrances (B) (Fig. 2A; Table S1) (${\chi }_{\mathrm{ageA/ B}}^{2}=21.39$, *df* = 1 *P* < 0.001). Although fewer animals and a higher proportion of adults were captured having crossed the tunnels (C) than those that reached tunnel entrances (B), these differences were not significant indicating that any movement bottleneck effect took place between fence and tunnel entrance movements but not subsequently (Fig. 2).

Overall, capture rates for *T. cristatus* adults and juveniles were not consistent through the mitigation system (Fig. 2A; Table S1). More adults were recorded after successful tunnel crossings (C) (55.7%) while juveniles were most frequently caught at the tunnel entrances prior to crossing (B) 55.4% (${\chi }_{\mathrm{adults}}^{2}=76.44$, *df* = 2 *P* < 0.001) (Fig. 2A; Table S1).

*L. vulgaris* displayed a different pattern to *T. cristatus*, although the low number of captures at all three locations makes it difficult to draw clear inferences (Fig. 2B; Table S1). Captures of both adults and juveniles of this species differed significantly among the three capture points in the system (${\chi }_{\mathrm{ageB/ C}}^{2}=4.90$, *df* = 1, *P* = 0.03; ${\chi }_{\mathrm{ageB/ C}}^{2}=6.60$, *df* = 1, *P* = 0.01) with fewest adults and most juveniles caught at stage B (${\chi }_{\mathrm{adults}}^{2}=10.97$, *df* = 2 *P* < 0.01).

There were no significant differences in *T. cristatus* sex-ratios concerning points A, B and C. However, the proportion of *T. cristatus* females captured was much higher than males overall (80.5% females) and at each point in the system (Fig. 2A; Table S1): 78.6% (A), 87.5% (B) and 85.2% (C). In contrast, *L. vulgaris* males were more frequently caught than females overall although this pattern was not consistent across the different capture points in the mitigation system: 66.7% males (A), 20.0% (B) and 60.0% (C) (${\chi }_{\mathrm{males}}^{2}=34.6$, *df* = 2, *P* < 0.001) (Fig. 2B; Table S1).

### Temporal and directional patterns of newt movements

*T. cristatus* captures at the fence (A) and tunnel (B, C) varied considerably among years (*H* = 117.75, *df* = 2, *p* < 0.001). MI_*y*_ values for A ranged from 0.33–4.00 captures per night, for B from 0.13–0.58 captures per night and for C from 0.08–0.77 (Table 3). *L. vulgaris* capture rates differed significantly among years (*H* = 26.17, *df* = 2, *P* < 0.001) although variance was relatively consistent between capture points (Table 3).

**Table 3:** Captures movement index (MI_*y*_) at each point in the mitigation system over the study period for two newt species.

	A				B				C			
Years	*N*	*MI*	Mean ± SD	*s*^2^	N	*MI*	Mean ± SD	*s*^2^	N	*MI*	Mean ± SD	*s*^2^
*Triturus cristatus* (Great crested newt)												
2008	99	0.88	1.79 ± 1.62	2.62	66	0.58	0.43 ± 0.30	0.09	32	0.28	0.27 ± 0.20	0.05
2010	125	1.95			35	0.55			49	0.77		
2011	21	0.33			15	0.23			5	0.08		
2012	256	4.0			8	0.13			11	0.17		
*Lissotriton vulgaris* (Smooth newt)												
2008	5	0.04	0.12 ± 0.09	0.01	20	0.18	0.10 ± 0.09	0.01	11	0.10	0.08 ± 0.10	0.01
2010	10	0.16			12	0.19			14	0.22		
2011	3	0.05			1	0.02			0	0		
2012	14	0.21			2	0.03			0	0		

From 2010 overall mean values for successful tunnel crossings (numbers of newts caught at B relative to C) dropped for both newt species (Table 3). This pattern was particularly evident for *T. cristatus* captures despite an increase in B values during 2011 (Table 3). Overall, MI_*y*_ values for successful crossings (C) were relatively low for with the exception of 2012, remaining below 0.77 captures per night for *T. cristatus* and below 0.22 captures per night for *L. vulgaris*, with zero crossings for the last two monitoring years for *L. vulgaris* (Table 3).

**Figure 3:**
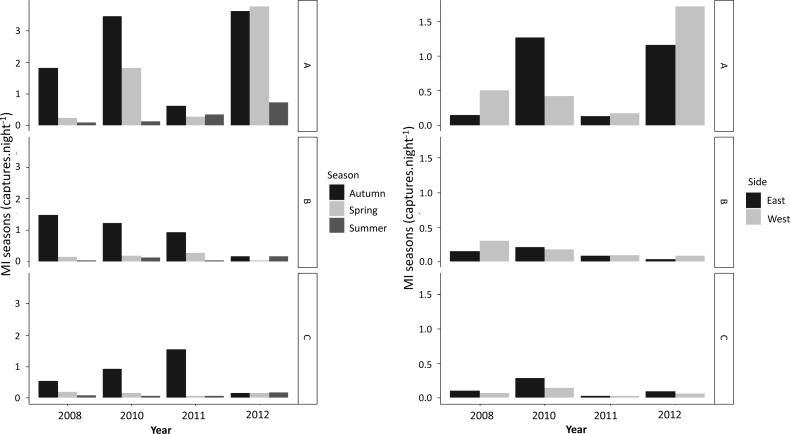
Season (MI_*s*_) and direction (MI_*d*_) patterns of MI values (captures.night^- 1^) for newt capture points (A, B and C) observed per year for both newt species.

More newts were captured during autumn than in any other season (Fig. 3). Overall, higher numbers of successful crossings were also recorded during autumn over the years, whereas spring and summer mitigation use was low (Fig. 3). GLMM analysis showed that seasonality had an effect on attempting and successful crossings for both species with no effect from age (Table 4).

**Table 4:** Parameter estimates for the generalized linear mixed-effect model (GLMM) with a zero-inflated Poisson (ZIP) of seasonal and directional movement indexes (MI_*s*_ = 144 and MI_*d*_ = 96) for both newts species (*T. cristatus* and *L. vulgaris*) with coefficient (*β*); standard error (SE); *t* statistics (*t*-value) and corresponding significance (*P*-value). Null deviance (ND) and residual deviance (RD) include information on predicted response by the null and by all predictors model, respectively.

Response	Intercept	SE	*Z*	*P*	AIC	Likehood ration
**Seasonality**						
**Null**	−1.35	0.53	−2.54	0.01		
**Global**	−1.49	0.58	−2.55	0.01	146.6	−64.30
TypeB	−1.23	0.40	−3.09	0.002		
TypeC	−1.55	0.45	−3.43	<0.001		
Spring	−0.99	0.36	−2.75	0.01		
Summer	−2.30	0.61	−3.72	<0.001		
Species	2.22	0.51	4.29	<0.001		
**type** + ** season** + ** species**	−1.55	0.56	−2.75	0.006	**144.8**	−64.38
**type** + ** season**	0.08	0.32	0.26	0.79	174.2	−80.12
**season** + ** species**	−2.24	0.55	−4.04	<0.001	160.3	−74.16
**Direction**						
**Null**	−2.24	0.59	−3.78	<0.001		
**Global**	−2.79	1.01	−2.76	0.005	68.1	−26.06
TypeB	−1.38	0.80	−1.71	0.08		
TypeC	−1.56	0.87	−1.80	0.07		
Species	2.11	0.96	2.19	0.02		
**type** + ** side** + ** species**	−2.99	0.98	−3.04	0.002	**66.7**	−26.34
**type** + ** side**	−1.46	0.47	−3.07	0.002	72.5	−30.25
**side** + ** species**	−3.71	0.96	−3.86	<0.001	68.4	−29.20

During the study period, movement of newts was recorded on both sides of the road in two directions (Fig. 3B). MI_*d*_ varied significantly between sides along the years, but with no prevalence of movement from any particular direction (Fig. 3B; Table 4). However, models showed potential effect of species in movement direction between sides (Table 4).

## Discussion

By quantifying the different types of movements (A, B and C) this study has shown how two pond-breeding amphibian species used road mitigation tunnels, demonstrating differences in attempted and successful crossings and movement along the system, while highlighting substantial temporal and directional variability.

### Newt movement patterns at the road mitigation system

The observed lower rates of successful and attempted crossings relative to fence movement suggest a movement bottleneck effect for dispersers. This is in line with other studies showing amphibian numbers reducing along the mitigation system (Patrick et al., 2010; Pagnucco, Paszkowski & Scrimgeour, 2012).

Newts require well-kept and well-designed fences to prevent them from climbing onto the road (Schmidt & Zumbach, 2008). The substantially higher capture rates at fences compared to the tunnels could be explained by the “fence effect”: Allaback & Laabs (2003) showed that salamanders attempted to avoid road mitigation fences and once avoided they appeared disoriented and moved in other directions, not necessarily towards mitigation tunnels. In this respect, fences can mimic the barrier effects of roads (Hels & Buchwald, 2001; Jaeger & Fahrig, 2004; Mazerolle, Huot & Gravel, 2005), especially for newts, including *T. cristatus,* which are relatively poor dispersers overland (Jehle & Arntzen, 2000). Future research should investigate optimising fence and tunnel placement in order to minimise such potential barrier effects.

Sex ratios differed between capture points along the mitigation system for both newt species but overall far more females than males were observed for *T. cristatus*, a pattern previously recorded for some salamander species (Aresco, 2005; Pagnucco, Paszkowski & Scrimgeour, 2012). This may be due to differences in: (1) population sex ratio, (2) sex differences in time spent in the pond and (3) migration distances to and from the ponds (Latham & Knowles, 2008; Hayward, 2002; Schabetsberger et al., 2004; Jarvis, 2012). Newt home ranges are generally small (with linear movements away from the pond between 30–400 m) (Jehle, 2010; Jehle & Arntzen, 2000; Müllner, 2001) but adult females undertake longer distance movements in autumn compared to males in the closely related Italian crested newt—*Triturus carnifex* (Schabetsberger et al., 2004). The differences in migratory behaviour between male and female newts and both pond position and distance to the tunnel may influence amphibian cues and motivation to move and consequently, the capture rates along the system (Buck-Dobrick & Dobrick, 1989; Sinsch, 1990). Our results suggest that the sex-biased migratory behaviour and the considerable distance from trapping points to the nearest ponds (30–74 m) favoured females over males, at least for *T. cristatus.*

The potential impact on the reproductive success of the population from the low adult male crossing rates observed in this study remains unknown but might be compensated by juvenile dispersal assuming no sex bias in crossing rates for this category. However, while the high juvenile movement in autumn indicates these movements as dispersal, the ultimate reasons for adult female long distance travel in autumn, including crossing through the road tunnels, remain unclear and somewhat contradict studies showing high breeding site fidelity for adults of this species (Jarvis, 2012).

Moreover, variability in movement is also linked to differences in behaviour of individuals, and this is true for different populations (Sinsch, 2014). The spatial context of the individual will define and trigger its decision to move or to stay (Baguette & Dyck, 2007). However, studies on individual-based spatial behaviour of newts to improve mitigation systems are still inexistent. Results could clarify how mitigation structures influence temporal and permanent residency of newts in the vicinity of the mitigation, fundamental for quantifying patterns of terrestrial movement and connectivity for broader scales (Baguette & Dyck, 2007; Baguette et al., 2013).

### Annual patterns of newt movements

Usage rates by newts of the mitigation system varied considerably among years. Captures at the fence increased over time while captures at the tunnel entrances and subsequently, the actual crossings, decreased. This could reflect how environmental variables influence movements of newts throughout the system. Favourable environmental conditions will not only facilitate amphibian dispersal across the landscape but also influence frequency of migrations (Sinsch, 1990; Sinsch, 2014). We suspect that the prolonged dry weather conditions in 2011 could have contributed to the decline in fence captures in 2011 and attempts and successful crossings in 2012. However, studies showing evidence of unfavourable weather conditions influencing tunnel environments and consequently newt behaviour have not been documented to our knowledge.

Low rates of successful crossings in tunnels were also reported before for Salamandridae family: *L. vulgaris* in Germany (12% of the attempted crossings) (Brehm, 1989), *Ambystoma macrodactylum* and *Taricha granulosa* (4% each) (Malt, 2012), *Ambystoma macrodactylum croceum* (9% of those detected at the fence; Allaback & Laabs, 2003) and 1%–23% for the same species in Canada (Pagnucco, Paszkowski & Scrimgeour, 2012) over a 2–3 year period. The higher crossing success rate in our study was potentially caused by the very large diameter of the tunnels compared to other studies, although at 30 and 40 m these tunnels are amongst the longest ever used for amphibians.

### Seasonal movements

Tunnels were mostly used for autumn movements, which for *T. cristatus* are typically long-distance (Jehle & Arntzen, 2000). An increase in tunnel use by juveniles during this season might be linked to the start of the postmetamorphic phase and emergence from ponds (Duff, 1986; Hayward, 2002) as well as juvenile dispersal attempts. Spring movement rates for adults were low, indicating that breeding migration (from terrestrial hibernation sites to aquatic breeding habitats) through tunnels was very limited. This may be due to a combination of factors such shorter-distance movements by adults during spring migration to breeding sites (Griffiths, Sewell & McCrea, 2010; Jehle & Arntzen, 2000) and potential overwinter mortality.

As part of the ongoing site monitoring the 200 m road section above and near the mitigation tunnels was surveyed intensively on foot every two early mornings for 220 days in the maximum activity period for amphibians, between September 2013 and October 2014. No amphibian road kill was ever recorded despite the fact that newts, especially *T. cristatus,* were occasionally seen near the road surface at night. The lack of observed amphibians road use could indicate the effectiveness of the fence system for mitigating road mortality (Cunnington et al., 2014). However, the road may also represent a significant barrier to movement contributing to a possible display of avoidance behaviour (Mazerolle, Huot & Gravel, 2005).

Prior to the construction of the road and mitigation structures, a two year study was conducted to better understand connectivity and movement behavior of *T. cristatus* in the southern part of the same site (HCI, 2006). The results indicated a very similar pattern compared to the newt movement observed in our study. The vast majority of the newts were trapped in autumn, with very little adult pond migration movement in spring for *T. cristatus*. Equally, overall there was a smaller number of males compared to females and large differences in the number of individuals between the two years of surveys (HCI, 2006). The similar patterns with pre-road construction movement data for this species suggest that the tunnel mitigation system may partially influence the newt movements but the general patterns remain unchanged.

### Movement directionality in the mitigation system

Directionality of movements differed between the two species and years of monitoring. *T. cristatus* showed higher movement rates from the large area of habitat in the west to the smaller area in the east while *L. vulgaris* mostly moved from east to west, balancing the use of the mitigation between the two sides during years of captures. Although the precise drivers of differential direction of movement are unclear, the extensive suitable habitat on both sides of the road makes it unlikely that movement through tunnels is driven by habitat availability. It is possible that density-dependent dispersal from the larger *T. cristatus* population in the west is responsible for the observed pattern in this species, but the opposite pattern in *L. vulgaris* is less easily explained. However, our results are consistent with the importance of winter and breeding habitats on both sides of the mitigation system for intra-population movements (Oldham et al., 2000; Malmgren, 2002; Hartel et al., 2010). Studies from mitigation schemes with unidirectional movement between seasons (breeding ponds on one side of the mitigation scheme, terrestrial, non-breeding habitat on the other) indicated lower, adult-biased numbers of amphibians crossing (Pagnucco, Paszkowski & Scrimgeour, 2012; Allaback & Laabs, 2003). In order to provide adequate connectivity over sub-populations over time, tunnels should facilitate movements of amphibians in both directions and for both adults and juveniles. Nevertheless, the dynamics of *T. cristatus* sub-populations, including adult survival, are driven mainly by juvenile dispersal, and effective recruitment can increase the probability of successful breeding (Griffiths, Sewell & McCrea, 2010). In this case, the larger population can be considered the source and the mitigation measure may play an important role in maintaining population viability.

### Implications for conservation and conclusions

Understanding how functional connectivity and population movements are influenced by road mitigation infrastructure could underpin the development of improved mitigation schemes.

The very low adult tunnel crossing rate by newts in spring raises fundamental questions about how such mitigation systems should be implemented for newt species. Road tunnels for newts may maintain landscape connectivity through facilitating autumn dispersal but whether or not it supports spring migration to breeding sites where a road separates terrestrial and aquatic habitat remains unclear.

Although road mitigation projects can be focused on single species, as in this case for *T. cristatus,* wider species impact monitoring would be required for a better understanding of the mitigation impacts. This should include potential predators, competitors, other protected species or pest species.

We observed considerable annual variation in captures and successful crossing rates, highlighting the need for long-term monitoring both to assess the effectiveness of individual mitigation schemes in maintaining connectivity. The 5 years of monitoring carried out in our study, and which are typically required in the UK, are probably an absolute minimum to adequately do this. Moreover, our results underline the value of improving the evaluation of terrestrial movements for newt species in order to successfully mitigate the negative population impacts of road networks.

##  Supplemental Information

10.7717/peerj.2922/supp-1 Table S1 *T. cristatus* and *L. vulgaris* annual captures

10.7717/peerj.2922/supp-2 Table S2 GLMM random effect summary

10.7717/peerj.2922/supp-3 Supplemental Information 1 Species database

## Additional Information and Declarations

### Data Availability

The following information was supplied regarding data availability: The raw data has been supplied as a  Supplementary File.
